# Implementation strategy fidelity evaluation for a multidisciplinary Chest Injury Protocol (ChIP)

**DOI:** 10.1186/s43058-021-00189-8

**Published:** 2021-08-10

**Authors:** Sarah Kourouche, Kate Curtis, Belinda Munroe, Michael Watts, Sharyn Balzer, Thomas Buckley

**Affiliations:** 1 Faculty of Medicine and Health, Sydney Nursing School, The University of Sydney Susan Wakil School of Nursing and Midwifery, 88 Mallet St, Camperdown, NSW Australia; 2 Director of Critical Care Research, Illawarra Shoalhaven Local Health District, Warrawong, Australia; 3 Illawarra Health and Medical Research Institute, University of Wollongong, Wollongong, NSW Australia; 4 Emergency Services, Illawarra Shoalhaven Local Health District, Wollongong Hospital, Crown St, Wollongong, NSW Australia; 5 Intensive Care, Illawarra Shoalhaven Local Health District, Wollongong Hospital, Crown St, Wollongong, NSW Australia; 6 Emergency Services, Shoalhaven Memorial District Hospital, Shoalhaven, NSW Australia

**Keywords:** Implementation strategy, Intervention fidelity, Process evaluation, Behaviour change, Emergency, nursing, Injury, Blunt chest wall injury, Care bundle

## Abstract

**Background:**

Blunt chest wall injuries can lead to complications, especially without early intervention. A blunt *Ch*est *I*njury *P*rotocol (ChIP) was developed to help improve the consistency of evidence-based care following admission to the emergency department. Implementation strategy fidelity is the extent to which the strategies of implementation are delivered in line with the intended plan. The aim of this study was to assess fidelity to the strategies of the implementation plan developed for ChIP.

**Methods:**

A retrospective evaluation of strategies used for implementation was performed, specifically the behaviour change techniques (BCTs). BCTs were used as part of an implementation plan derived based on the Behaviour Change Wheel from results from a staff survey at two hospitals.

Levels of implementation or adaptation for BCTs were scored by implementers as follows: ‘Were the behaviour change interventions implemented?’ (0 = ‘not implemented’, 1 = partially implemented, and 2 = fully implemented); ‘Were adaptations made to the implementation plan?’, scored 1 (many changes from plan) to 4 (just as planned). Free text explanation to their responses was also collected with supporting evidence and documentation (such as emails, implementation checklists, audit reports, and incident reports).

**Results:**

There was high overall fidelity of 97.6% for BCTs partially or fully implemented. More than three quarters (32/42, 76.2%) of the BCTs were fully implemented with an additional 9/42 (21.4%) partially implemented. BCTs that were not fully implemented were social support, feedback on behaviour, feedback on outcomes of behaviour, adding objects to the environment, and restructuring the environment. The modes of delivery with poorer implementation or increased adaptations were clinical champions and audit/feedback.

**Conclusions:**

This study describes the evaluation of implementation strategy fidelity in the acute care context. The systematic use and application of the behaviour change wheel was used to develop an implementation plan and was associated with high implementation strategy fidelity. A fidelity checklist developed during the implementation process may help implementers assess fidelity.

**Trial registration:**

Trial registered on ANZCTR. Registration number ACTRN12618001548224, date approved 17/09/2018

**Supplementary Information:**

The online version contains supplementary material available at 10.1186/s43058-021-00189-8.

Contributions to the literature

- Implementation research should include focus on evaluations of fidelity including evaluation of the implementation plan to improve the internal and external validity of implementation. Implementation strategy fidelity evaluates the extent to which implementation strategies were used to help determine the relationship between the implementation plan and success of the intervention.
- Retrospective review is possible for implementation strategy fidelity evaluation in the acute care context.
- The findings in this study contribute to recognised gaps in the literature, including how implementation researchers should address implementation strategy fidelity.

## Background

Blunt chest wall injury, such as fractured ribs, can lead to serious health complications like pneumonia if not managed appropriately [1]. To improve the consistency of care for patients with blunt chest wall injury and minimise their risk of complications, an evidence-informed protocol was developed [2]. This protocol incorporated the best available evidence, included an early notification mechanism, and guided clinicians on management. Implementation strategies were developed using the Theoretical Domains Framework and the Behaviour Change Wheel (BCW) [3] to facilitate implementation at two hospitals in regional Australia [4]. The BCW is a framework based on behaviour change theory that can help address clinician behaviour change [3]. Using theoretical frameworks is important, especially for a high acuity environment such as the emergency department with multidisciplinary teams [5]. Though the use of a framework improves the quality of the implementation strategies, evaluation of the fidelity of implementation is required to determine the relationship between the implementation plan and the success of the intervention [6].

Implementation strategy fidelity is the extent to which implementation strategies were used [7]. This contrasts with the fidelity of delivery (or treatment fidelity), which is the extent to which an intervention is delivered in line with the intended plan [7–9]. Fidelity of delivery is an evaluation of what was delivered clinically, whereas implementation strategy fidelity evaluates how the plan was delivered [10]. The delineation is often unclear in the literature and only referred to as implementation fidelity which encompasses both the fidelity of delivery and implementation strategy fidelity [11].

Generally, evaluation of implementation fidelity allows for comparisons between studies and allows for replication by ensuring that implementation is occurring as intended [9]. Differences in delivery can impact the effects of an intervention between sites, and fidelity can help control for this [12]. Fidelity is critical to the internal and external validity of implementation [7]. Fidelity is important for exploring the mechanisms through which change occurs [13].

Despite its importance, evaluation of implementation fidelity is rarely reported within intervention trials [10]. A survey of complex healthcare interventions identified that frameworks were not used by almost 70% of implementers in research reporting [8]. Some have suggested that this may be due to limited comprehensiveness of frameworks and language for evaluating implementation fidelity [7, 14]. Furthermore, there is little consensus on how to assess for fidelity [7].

There have been multiple frameworks suggested for implementation fidelity in the implementation literature. Among these, five main concepts have been described for implementation fidelity: adherence to an intervention, dose or exposure, programme delivery, participant engagement, and programme differentiation [9, 15, 16]. However, there is also contention as to whether these all are needed for fidelity evaluation [17]; furthermore, some are not relevant to the acute care setting. The aim of this study was to assess fidelity to the strategies of the implementation plan developed for ChIP. This paper is part of a larger study evaluating ChIP for patient, health service, and cost outcomes and for fidelity of delivery [18]. This is in the manuscript preparation stage. The fidelity of delivery has been evaluated separately [19]. The treatments within the protocol are all evidence-based and reflect other existing pathways that have been demonstrated to be effective [20].

### The intervention

**Fig. 1:**
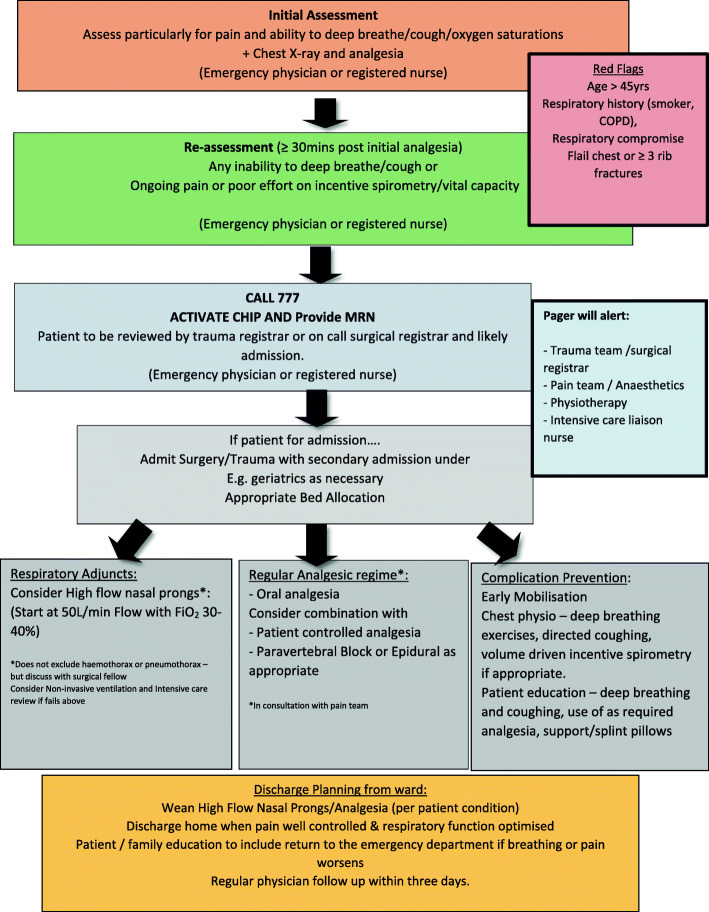
The blunt chest injury care bundle (ChIP)

A care bundle was developed through an integrative review of chest injury interventions from 81 studies [2]. Care bundles are an intervention based on three to five key interventions combined [21]. The blunt chest injury care bundle was based on four main pillars of care: respiratory adjuncts, regular analgesia regime, complication prevention, and surgical fixation tailored to each patient within the context of their presentation [2]. The care bundle was adapted for the sites as surgical fixation was not possible due to a lack of trained staff and operating resources (Fig. 1). The blunt chest injury care bundle was called ‘ChIP’ (Chest Injury Protocol) for implementation for ease in remembering and use.

ChIP was designed for use by the multidisciplinary team and activated in the emergency department (ED). ChIP provided a guide for assessing the patient with blunt chest injury in the ED and a process for activation of an early notification system. The relevant clinicians were then expected to review the patient within 60 min and initiate care tailored to the individual. The responding clinicians included physiotherapy; the surgical, intensive care, pain teams; and, if required, the general medicine or geriatric team. After hours, the response required was slightly different. While the surgical, pain, and intensive care teams still reviewed the patient, the physiotherapist and aged care team reviewed the patient as a priority the following day.

The ChIP policy was opened for review and comment to the local health district. Any staff from the local health district could comment and give feedback on ChIP. Furthermore, direct feedback was sought on the intervention from staff and departments involved in the implementation. As a result, minor changes were integrated, such as adding pager numbers and contacts.

### The implementation plan

Implementation of ChIP commenced 22 November 2017 using an implementation plan developed using the BCW [3]. In previously published work, authors surveyed staff from 12 departments across two sites to identify facilitators and barriers to behaviour change [22]. The survey was informed by the Theoretical Domains Framework and identified nine facilitators and six barriers to implementation across eight domains [22]. An eight-step process based on the BCW was followed. The facilitators and barriers from the survey were mapped to intervention functions, behaviour change techniques (BCTs), and modes of delivery [22]. These were used to identify a mechanism of action often referred to as implementation strategies in the literature. BCTs are the active ingredients of change provided in a theory-linked taxonomy [6] and are defined within a taxonomy [23]. The BCTs can act as a coding framework for evaluation [24].

Each step was reviewed against the Affordability, Practicability, Effectiveness and cost-effectiveness, Acceptability, Side-effects or safety and Equity (APEASE) criteria [6]. Intervention functions are ‘broad categories by means of which an intervention can change behaviour’, and modes of delivery are the mediums through which the BCTs are delivered, such as media or guidelines [6]. The mechanisms of action combine the BCTs and modes of delivery into an actionable step. The complete implementation plan is available as an additional file [see Additional file 1]. Due to the high theoretical basis, following the implementation plan and the strategies outlined in the plan should result in higher fidelity and sustainability of the ChIP intervention. Strategies such as staff appearing in the ChIP video were intended to enable high fidelity and acceptability of implementation (Table 1) [25].

**Table 1:** Behaviour change techniques (including their relevant numbers from the taxonomy), intervention functions, modes of delivery (bold), examples of the mechanisms of action, expected dose, and intended implementation outcome

BCTs	Intervention functions	Mechanism of action	Target barrier(s)/facilitator(s) (TDF domain)	Expected dose	Intended implementation outcome
1.9. Commitment	Incentivisation, enablement	Staff appear in a **video** promoting ChIP	Remembering to use protocol (memory, attention, and decision processes)	At least one staff member from each of the disciplines involved	Increase fidelity/acceptability from all disciplines
2.2. Feedback on behaviour	Education, persuasion, incentivisation	Staff compliance monitored through **audits** and staff will be informed of the results informally by **clinical champions**, through **newsletters** and **emails**	Belief of consequences of care bundle (belief about consequences) Understanding of evidence-informed interventions for a patient with blunt chest injury (knowledge)	Audits to be done monthly for 12 months	Increase fidelity
2.7. Feedback on outcome(s) of behaviour	Education, incentivisation, training	Feedback given to staff from **audit** results on patients treated with the care bundle through **clinical champions**	Understanding of evidence-informed interventions for a patient with blunt chest injury (knowledge) Belief of consequences of care bundle (belief about consequences) Emotions relating to commencing new protocol (emotion) Remembering to use protocol (memory, attention, and decision processes) Confidence in patient assessment skills (physical skills)	For audits after 1, 2, and 3 months then if issues	Increase sustainability
3.1. Social support	Enablement	**Clinical champions** chosen from each area receive extra training to be able to provide extra support	Social supports (social influences)	2 clinical champions from each ED	Increase fidelity
4.1. Instruction on how to perform behaviour	Training	Staff receive instructions on behaviour via a **video, educational sessions**, and **clinical champions**	Confidence in patient assessment skills, confidence in skills needed for evidence-informed management of blunt chest injury, adequate skill in regional analgesia prescription and management (physical skills) Remembering to use protocol (memory, attention, and decision processes)	Education sessions to be attended by staff	Increased fidelity
5.1. Information about health consequences	Education, persuasion	Staff informed about the improvement in pneumonia rate reduction with the protocol from the previous study through **video**, **educational sessions**, **flyers**, **newsletters**, and **emails**	Belief of consequences of care bundle (belief about consequences) Understanding of evidence-informed interventions for a patient with blunt chest injury (knowledge)	All relevant staff	Increased acceptability
6.1. Demonstration of behaviour	Training, modelling	Staff receive demonstrations of behaviour via a **video**, **educational sessions**, and **clinical champions**	Confidence in patient assessment skills, confidence in skills needed for evidence-informed management of blunt chest injury, adequate skill in regional analgesia prescription and management (physical skills) Remembering to use protocol (memory, attention, and decision processes) Identify with professional role associated with care of blunt chest injury patients (professional/social role and identity) Emotions relating to commencing new protocol (emotion)	All relevant staff	Increased fidelity
6.3. Information about others’ approval	Education, persuasion	Local staff appear in the ChIP **video** showing support	Identify with professional role associated with care of blunt chest injury patients (professional/social role and identity)	Senior staff from each discipline	Increase acceptabilty
7.1. Prompts/cues	Education, environmental restructuring	A visual prompt (**screen icon**) developed for the electronic medical record to flag to staff that patient is eligible for care bundle	Remembering to use protocol (memory, attention, and decision processes)	On computer system	Increase fidelity and sustainability
		**Flyers** put up around the workplace to remind staff of the care bundle		In both EDs, ICUs, trauma wards	Increase fidelity
8.3. Habit formation	Training	Staff encouraged to assess all potentially eligible patients systematically in the **video** and **educational sessions**	Confidence in patient assessment skills, confidence in skills needed for evidence-informed management of blunt chest injury, adequate skill in regional analgesia prescription and management (physical skills) Remembering to use protocol (memory, attention, and decision processes)	Staff from relevant disciplines	Increase fidelity
9.1. Credible source	Persuasion	Senior local **staff** appear in a **video** informing staff about and promoting ChIP	Identify with professional role associated with care of blunt chest injury patients (professional/social role and identity)	At least one staff member from each discipline	Increase acceptability
12.1. Restructuring the physical environment	Environmental restructuring, enablement	**Equipment** necessary for ChIP placed in a location that ensures ease of access	Remembering to use protocol (memory, attention, and decision processes) Access to protocol, the protocol design, access to equipment (environmental context and resources) Emotions relating to commencing new protocol (emotion)	HFNC machines and incentive spirometry in ED	Increase fidelity
		**Equipment** adequately labelled with instructions		All new HFNC machines	Increase fidelity
		Additional **equipment** supplied to ensure adequate supply (high-flow machines and incentive spirometry)			
		ChIP tested by staff to ensure ease of use		Tested once prior to go live	Increased fidelity
12.5. Adding objects to the environment	Environmental restructuring, enablement	An **icon** added for the electronic medical record to flag to staff that patient is eligible for care bundle	Remembering to use protocol (memory, attention, and decision processes) Access to protocol (environmental context and resources) Emotions relating to commencing new protocol (emotion)	Add prior to go live	Increased fidelity
		A **pager** setup to be able to contact staff responding to ChIP		Set up prior to go live	Increased fidelity
13.1. Identification of self as a role model	Persuasion, enablement	Staff asked to volunteer for the roles of **clinical champions** and to be in the video	Emotions relating to commencing new protocol (emotion) Identify with professional role associated with care of blunt chest injury patients (professional/social role and identity)	Champions at each site	Increased fidelity and sustainability
15.1. Verbal persuasion about capability	Persuasion, enablement	Staff encouraged during **educational sessions** and by **clinical champions** that they are capable of following ChIP	Emotions relating to commencing new protocol (emotion)	At each session	Increased fidelity and acceptability

Definitions for intervention functions: *Education*: increasing knowledge or understanding, *Persuasion*: using communication to induce positive or negative feelings or stimulate action, *Incentivisation*: creating an expectation of reward, *Training*: imparting skills, *Environmental restructuring*: changing the physical or social context, *Modelling*: providing an example to aspire to, *Enablement*: increasing means/reducing barriers

The implementation plan was discussed with stakeholders in a series of meetings at both sites. Implementers met with the managers for each department involved and presented the plan at education meetings to get feedback. It was reviewed and approved by key implementation team members and stakeholders. The stakeholders included business sponsors, executive sponsors (such as critical care directors, directors of nursing, physiotherapy district manager), trauma committee, end users, and managers from surgical, intensive care, ED, general medicine, geriatrics, and physiotherapy.

The implementation plan was designed to target BCTs and intervention functions through strategic mechanisms of action to address facilitators and barriers. The main modes of delivery for these mechanisms of action were a video featuring staff from the sites promoting ChIP, face-to-face educational sessions, prompts including an icon on the electronic medical record, other advertising such as flyers and emails, clinical champions, and audit/feedback. Resources were prepared and developed for each of these areas before implementation by the implementation team. The name ChIP served well as a theme of implementation, for example, putting potato chips near flyer advertisements for ChIP and using packets of chips (British—crisps) as the icon for the electronic medical record. A timeline of preparation and implementation is in Table 2.

**Table 2:** Implementation of ChIP timeline, monitoring, and stakeholder roles

Date	What	Who
18 October 2017	Policy online as draft for comment	ED CNC
20 October 2017	Research nurse hired	ED CNC/implementation team
23 October 2017	High-flow nasal cannula (HFNC) machines labelled with instructions	ED CNC
October 2017	Filming for video by the implementation team	Implementation team, staff from departments involved
25 October 2017	Item prepared for surgical staff newsletter	ED CNC/implementation team
26 October 2017	Video Version 1	Implementation team
30 October 2017	Video Version 2 Training materials (including PowerPoint) developed Chip packets for staff tearoom Notices prepared for newsletter	Implementation team
31 October 2017	Organise dates and instructors for educational sessions for - Ward - ICU - ED - Surgical registrars - Anaesthetic - Physiotherapists	Implementation team
6 November 2017	Implementation plan final Draft emails for each department organised Advertising flyers prepped	Implementation team
7 November 2017	Final version of ChIP video completed and uploaded to YouTube channel	Implementation team
7 November 2017	Email went out to all staff, managers Except site 2	Implementation team/managers
13 November 2017	Premature ChIP activation at site 1, went smoothly	ED staff
15 November 2017	Incentive spirometers ordered	ED CNC
16 November 2017	Discussion re role of ICU liaison: - Education for ward, switch, and bed manager - Audit switch pagers and check compliance - Ensure education for new surgical/ICU and anaesthetic registrars is sustainable, will incorporate ChIP into hospital orientation sessions.	Implementation team
20 November 2017	Stakeholders approve policy Organise educational sessions to be delivered	Stakeholders—executives, managers, etc. Fisher & Paykel Implementation team
21 November 2017	11 new pagers programmed and tested with all of their existing capcodes plus the new capcode allocated for 880	Implementation team
22 November 2017	**Go live date**	
22 November 2017	First patient has ChIP activated at site 2 (without electronic medical record being active)	
23 November 2017	Consult Clinical Pathway order for ChIP is available in FirstNet for site 2	
23 November 2017	Contract for casual RN (clinical champion) processed	
27-28 November 2017	Based on feedback: - PowerPoint presentation edited to include how to chart HFNP - Videos updated—both updated to include patient education, incentive spirometer, how to chart HFNC - A dedicated ‘cheat sheet’ algorithm for site 2 as their notification process is different	Implementation team
30 November 2017	Queries from surgical staff regarding charting of HFNC and observations	
4 December 2017	15 educational sessions more provided this week ChIP in nursing grand rounds this week Some issues with HFNC being charted to medication chart—education regarding same	Implementation team
5 December 2017	Switched incentive spirometers to volume-driven devices	Physiotherapy/implementation team
1 February 2018	44 ChIP notifications since November	
6 February 2018	Fact sheet for patients and contacts at site 2 added to electronic medical record (emailed 27/11/17)	Implementation team

*Abbreviations*: *ChIP* Chest Injury Protocol, *CNC* clinical nurse consultant, *ED* emergency department, *eMR* electronic medical record, *ICU* intensive care unit

The resources developed by the intervention developers prior to implementation commencement and the plans for the modes of delivery per the implementation plan are now more fully described per the behaviour change taxonomy [23] with further detail as recommended by Proctor et al. [26]. These are summarised in Table 1, including the barriers and facilitators that guided the inclusion [22]. The results evaluate these plans.

#### Modes of delivery to operationalise the implementation plan

##### Video

**Fig. 2:**
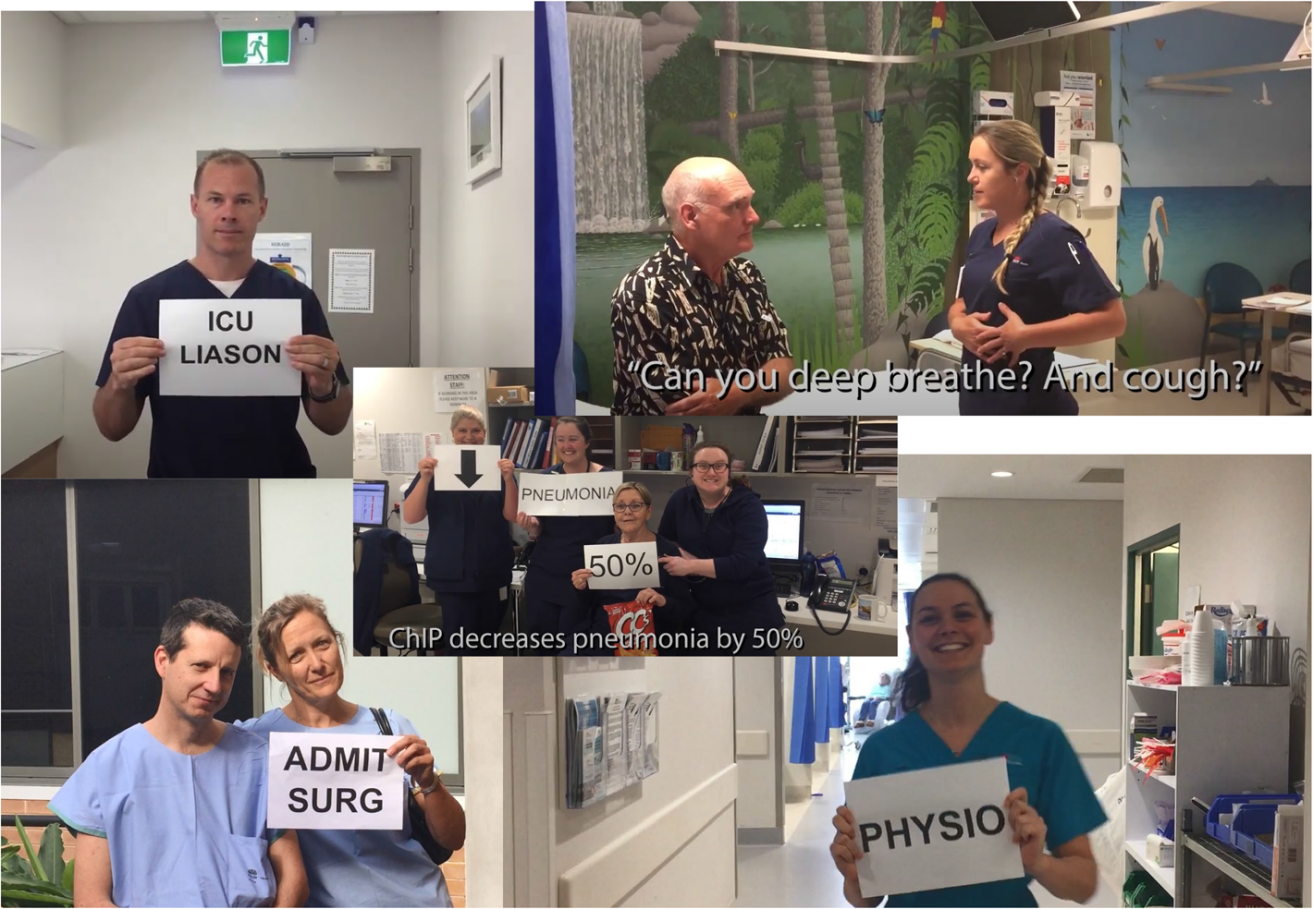
Collage of stills from the ChIP video

A video was developed to address six BCTs and the intervention functions of incentivisation, enablement, training, education, persuasion, and modelling. The intervention development team produced the video following a simple storyboard. It featured multidisciplinary junior and senior staff to show the commitment and approval across the board to ChIP. The video featured demonstrations of the instructions that were required by the staff when implementing ChIP, modelling the behaviour required. The footage was filmed and edited by the implementation team using a smartphone and accessible video software. The video was uploaded to a private YouTube account for easy access by the implementation team. The planned dose was for the video to be shown during educational sessions, targeting each of the disciplines involved and added to all communications, including emails for the duration of the implementation period and new staff. The video was short, at less than 2 min long. The footage took staff through an example of using ChIP and the intervention options available featuring staff from various roles (Fig. 2). The video used can be viewed here https://youtu.be/VlMz1PjzmBk.

##### Visual prompts

Visual prompts were developed for the ChIP rollout addressing three BCTs and intervention functions of education, persuasion, enablement, and environmental restructuring. Flyers were developed for the wards and the ED; these would be placed around the departments before implementation commencement by the implementation team. Email drafts were developed for the various departments involved by the implementation team. Patient flyers were also developed for patients who had received a ChIP call by the implementation team. Newsletter items were also prepared, adjusted based on where they would be displayed and who would be seeing them. They included eye-catching advertisements using a ‘ChIP logo’ of a packet of chips. Notices were prepared to be placed in the clinical communication books.

##### Facilitators/clinical champions

Facilitators/clinical champions addressed eight BCTs and intervention functions of education, persuasion, training, modelling, incentivisation, and enablement. The implementation plan fostered consistent messaging and implementation across sites. Facilitators played an essential role in the implementation plan as strong facilitators can drive implementation and enable change [27]. The ED clinical nurse consultants (CNCs) played the role of facilitator at the two sites. Discussions with facilitators at sites frequently occurred (several times per week) at peak times and less frequently (once a fortnight) during other times. The facilitators for the implementation of ChIP were part of the existing workforce of the hospitals and in positions where they would be able to facilitate the change as part of their roles. Furthermore, one of the strategies used within the implementation plan was to employ clinical champions at each site to improve the consistency of implementation. Clinical champions were to be employed by the emergency CNC using research funding for 3 months.

##### Audit and feedback

Audit and feedback addressed two BCTs and intervention functions education, persuasion, and incentivisation. Feedback during the implementation process was to be sought through formal and informal measures. Formal feedback measures included audits, incident reports, and morbidity and mortality meetings. Informal feedback measures included informal discussions with staff. The plan was for the intensive care unit (ICU) liaison CNC to respond to ChIP activation calls and provide informal feedback to the implementation team on the response to ChIP.

Audits would follow a template looking for failure in the process for missed ChIP calls or inadequate ChIP responses. They were to be conducted monthly for 12 months. Identified problems would be flagged so that adjustments could be made to the plan if necessary. The audits would be communicated back to the implementation team and department managers regularly.

##### Education sessions

Face-to-face education sessions addressed five of the BCTs and intervention functions education, persuasion, training, modelling, and enablement. The intervention development team prepared lesson plans and presentation slides for the educational sessions, which were adapted for the departments. The educational sessions were to be delivered to relevant staff by the ED CNCs and educators, ICU liaison for the wards, and senior physicians. All staff providing the educational sessions received the presentation slides and training from an investigator (KC), an ED CNC.

##### Environmental changes

Environmental changes addressed two BCTs and intervention function of environmental restructuring, education, and enablement. An icon was integrated into the electronic medical record software so staff from the emergency department could flag patients who had a ChIP activation. The icon was a chip packet the same as used on the advertisements.

High-flow oxygenation machines were secured through a grant from *Fisher & Paykel* and needed to be labelled with the ChIP policy and instructions for use. Incentive spirometry devices needed to be made available. Both of these items would need to be placed in easily accessible areas in the ED.

## Methods

This evaluation study assessed fidelity of the implementation strategies for a blunt chest injury care bundle (ChIP) based on BCTs used in an implementation plan. This evaluation focused on implementation strategy fidelity by the site implementation teams (authors BM, KC, SB, MW). Implementation team members at two hospital sites were asked to score BCT items to determine adherence to the implementation plan in the 30-months since the initial implementation.

This study is part of a larger study testing the efficacy of the ChIP. Research conducted as part of this study adhered to the National Statement on Ethical Conduct in Human Research by the Australian National Health and Medical Research Council [28] and was approved by the NSW Population & Health Services Research Ethics Committee (HREC/17/CIPHS/56). A STaRI (Standards for Reporting Implementation Studies) template has been completed along with this submission [see Additional file 2] [29].

### Context/setting

ChIP implementation was carried out at two hospital sites in regional NSW, Australia, within the same local health district. Site 1 is a regional trauma centre seeing approximately 70,000 presentations annually, and site 2 is a smaller district hospital with about 40,000 presentations to its emergency department annually [30].

### Data collection

To assess implementation strategy fidelity, each of the BCTs included as a part of the implementation plan, including the mechanism of action and mode of delivery, was retrospectively evaluated using a scoring system. This was assessed retrospectively to determine if what was planned by the implementation support team was implemented.

Four nurses responsible for leading implementation at the study sites (implementers) were questioned on the fidelity of the implementation plan with three implementers involved at each site. The implementers were the two district clinical nurse consultants (who were also familiar with intervention development at both sites), the clinical nurse educator of one the ED (site 2), and the ICU liaison who had also agreed to be a clinical champion (site 1). Implementers (KC, BM, MW, SB) individually completed an intervention fidelity scoring sheet electronically for each site (21 per site).

### Tool development

There is no consensus on the evaluation of implementation fidelity in the literature [7], though researchers have been working towards a solution [14]. The BCW recommends using the BCTs in the implementation fidelity evaluation [6], which was attempted for this study. Two questions were included for each of the mechanism of action within each BCT. The first question was ‘Were the behaviour change interventions implemented?’ This was answered using a three-point scoring system. A score of 0 meant the strategy was ‘not implemented’, a score of 1 indicated it was partially implemented, and a score of 2 indicated the strategy was fully implemented. This first question aimed to establish adherence or dose to the mechanisms of action from the implementation plan [9, 15].

A second question for each item was ‘Were adaptations made to the implementation plan?’ This question was scored 1 (many changes from the plan) to 4 (just as planned) [31]. This question aimed to establish fidelity (adaptation) of programme delivery [9, 15]. Adaptation is vital to the implementation process as implementers need to adapt to the context [31]. Therefore, it is important to keep track of the implementation process and the adaptations made.

Participant responsiveness was not addressed as behaviour change was targeted at staff rather than patients.

The implementers were asked to comment with supporting evidence and documentation. Examples of documentation included emails, implementation checklists, audit reports, and incident reports.

### Analysis

Data analysis was conducted by an independent researcher (SK) not involved in the onsite implementation. The analysis was performed using SPSS v25. Proportions were calculated for implementation and adaptation at each site and overall against each possible score. The quantitative data and supporting documentation data were compiled by an author (SK). The scores were compared by author (SK), and inconsistencies highlighted. The documentation sources were used to provide evidence for the score given and to evaluate discrepancies between scorers. It was also used to evaluate discrepancies between scorers. These inconsistencies were then discussed as a group where each implementer provided evidence as to why they gave their score. Discussions continued until consensus was reached. The documentation sources offered more information about the adaptations that were made.

## Results

Overall, more than three quarters (32/42, 76.2%) of the mechanisms of action were fully implemented, with an additional 9/42 (21.4%) partially implemented (Table 3). Only 1/42 (2.4%) of mechanisms were reported as not implemented. Almost a quarter of mechanisms required adaptations—slight adaptations 10/42 (23.8%) and 1/42 (2.4%) some adaptation.

**Table 3:** Behaviour change techniques, mechanisms of action in relation, and their reported implementation status and adaptations

BCTs	Intervention functions	Mechanism of action	Implementation status	Adaptations
1.9. Commitment	Incentivisation, enablement	Staff appear in a **video** promoting ChIP	Full	Nil
2.2. Feedback on behaviour	Education, persuasion, incentivisation	Staff compliance monitored through audits and staff will be informed of the results informally by **clinical champions**, through **newsletters** and **emails**	Partial	Minor
2.7. Feedback on outcome(s) of behaviour	Education, incentivisation, training	Feedback given to staff from **audit** results on patients treated with the care bundle through **clinical champions**	Full	Nil
3.1. Social support	Enablement	**Clinical champions** chosen from each area receive extra training to be able to provide extra support	Partial	Minor
4.1. Instruction on how to perform behaviour	Training	Staff receive instructions on behaviour via a **video**, **educational sessions**, and **clinical champions**	Full	Nil
5.1. Information about health consequences	Education, persuasion	Staff informed about the improvement in pneumonia rates reduction with the protocol from previous study through **video**, **educational sessions**, **flyers**, **newsletters**, and **emails**	Full	Nil
6.1. Demonstration of behaviour	Training, modelling	Staff receive demonstrations of behaviour via a **video**, **educational sessions**, and **clinical champions**	Full	Minor
6.3. Information about others’ approval	Education, persuasion	Local staff appear in the ChIP **video** showing support	Full	Nil
7.1. Prompts/cues	Education, environmental restructuring	A visual prompt (**screen icon**) developed for the electronic medical record to flag to staff that patient is eligible for care bundle	Full	Minor
		**Flyers** put up around the workplace to remind staff of the care bundle	Full	Nil
8.3. Habit formation	Training	Staff encouraged to assess all potentially eligible patients systematically in the **video** and **educational sessions**	Full	Nil
9.1. Credible source	Persuasion	Senior local **staff** appear in a **video** informing staff about and promoting ChIP	Full	Nil
12.1. Restructuring the physical environment	Environmental restructuring, enablement	**Equipment** necessary for ChIP placed in a location that ensures ease of access	Full	Nil
		**Equipment** adequately labelled with instructions	Partial	Minor
		Additional **equipment** supplied to ensure adequate supply (high-flow machines and incentive spirometry)	Partial	Minor
		ChIP tested by staff to ensure ease of use	Full	Nil
12.5. Adding objects to the environment	Environmental restructuring, enablement	An **icon** added for the electronic medical record to flag to staff that patient is eligible for care bundle	Full	Nil
		A **pager** setup to be able to contact staff responding to ChIP	Full at one site, not at all at second site	Major adaptation required at the small site
13.1. Identification of self as role model	Persuasion, enablement	Staff asked to volunteer for the roles of **clinical champions** and to be in the video	Full	Nil
15.1. Verbal persuasion about capability	Persuasion, enablement	Staff encouraged during **educational sessions** and by **clinical champions** that they are capable of following ChIP	Full	Nil

At site 1, 17/21 (81.0%) of mechanisms of action were agreed to have been implemented in full (implementation scores of 2), with 16/21 (76.2%) agreed to have been delivered as planned (adapt scores of 4). There were 4/21 (19.0%) that were reported partially implemented, and 5/21 (23.8%) that were reported to have slight adaptions from the plan (adapt score 3). At site 2, 15/21 (71.4%) of the BCTs were implemented in full, 5/21 (23.8%) were partially implemented, and 1/21 (4.8%) was not implemented.

Most BCTs were considered to be fully implemented. BCTs that were not fully implemented were social support, feedback on behaviour, feedback on outcomes of behaviour, adding objects to the environment, and restructuring the environment (Table 1). The modes of delivery associated with the BCTs that were not fully implemented, clinical champions, audit/feedback, and environmental restructuring, are discussed further as to their fidelity and adaptations.

### The video

Implementers agreed that the video was fully implemented across sites and was helpful. It was shown at the educational sessions and meetings and was also attached as a link in correspondence. Implementers reported that adaptations were made to the video to have two versions, one for each hospital site. Adaptations were also made to include the updated policy and include clips of how to prescribe high-flow oxygen as a treatment. Initially, there was some inconsistency in flow rates being administered.

### Facilitators/clinical champions

Implementers reported that clinical champions were partially implemented. They stated there were issues with the clinical champions at both sites. Some staff volunteered to be clinical champions, were given a temporary contract, and were orientated to the role by the clinical nurse consultant. However, from the eight staff hired and trained, only one staff member at each site continued to be involved in the implementation. The implementers stated that adaptations were attempted with several recruitment rounds. Nevertheless, only two clinical champions continued. The implementers reported that both of these staff performed well and provided support, though not the level of support that was planned. The clinical nurse consultants, clinical nurse educators, and medical leads further supported them in the role of clinical champion.

### Audit and feedback

Implementers at both sites reported audit and feedback were partially implemented. They reported that audit procedures were generated as per the implementation plan for the first 6 months post-implementation. During this time, the audits were run monthly using a standardised template and circulated to department heads. However, after this time, checks were less regular and only attended in response to feedback or random spot checks. The main concern from surgical and ICU registrars with the implementation was that there would be increased workload and the feedback helped allay these fears.

Implementers reported that feedback and audits were run in response to complaints or feedback. For example, a regular complaint received was that many patients were discharged from ED; however, reports were run demonstrating this happened 2% of the time, which was fed back to staff. Another example of feedback from ward staff was that the ED staff were not prescribing regular analgesia and were waiting for ICU or the pain service to chart prescriptions—leading to delays in analgesia delivery. To prevent this from continuing to occur, additional reinforcement of the role of the ED in charting analgesia was required, especially with the change in terms and rotations.

Another adaptation reported was that the audit process was incorporated into the role of the ‘between the flags’ CNC who already reviews cases of unplanned ICU admissions. This role has continued past the implementation team involvement.

### Environmental changes

Implementers reported some issues and need for adaptations with the implementation of some environmental changes. For example, implementers reported that though the high-flow oxygenation machines were labelled with the ChIP policy, this was insufficient to the implementation as they should have also been labelled with information for the use of the machines.

Implementers reported that adaptations were made to the type of incentive spirometers ordered per requests from the physiotherapy department. Implementers reported that physiotherapists preferred to use volume-driven incentive spirometers which ED did not have in stock. As the volume-driven spirometers were an evidence-based option, the new spirometers were ordered.

At the smaller hospital, implementers reported significant changes to the implementation plan for the paging system. It was identified that the majority of the responders did not use their pagers which was not identified during the barrier identification phase. Pagers were not used for a notification system as it was found that the responders did not carry them. The system had to be changed so that the ED staff would put an order through the electronic medical system. Activating it in this way gave activating staff a list with contacts to call.

An example of an adaptation led by participants was from the ortho-geriatric team who were concerned ChIP patients were being referred to them overnight by the emergency department but then not handed over to them in the mornings. The team came up with a solution, which they bought to the implementation team. They wanted to keep the pager in a common area where it could be easily accessed after a night shift with a sign reminding staff of the criteria.

## Discussion

This study describes the evaluation of implementation strategy fidelity in the acute care context. Implementation studies should include evaluation of implementation fidelity to avoid a type III error, where we may assume an intervention does not work, but it was not implemented effectively [32]. Methods for evaluation of implementation strategy fidelity are still in development [7]. Successful translation of evidence into practice requires effective mechanisms of action or implementation strategies; however, these have been inconsistently reported in the literature. There are multiple taxonomies for developing implementation strategies in the literature, making consistency difficult [23, 33]. A strength of this study was that the strategies used were theory-driven, and these were used to inform the evaluation [13]. Subsequently, the theory-driven mechanisms of action were used to inform the evaluation.

**Fig. 3:**
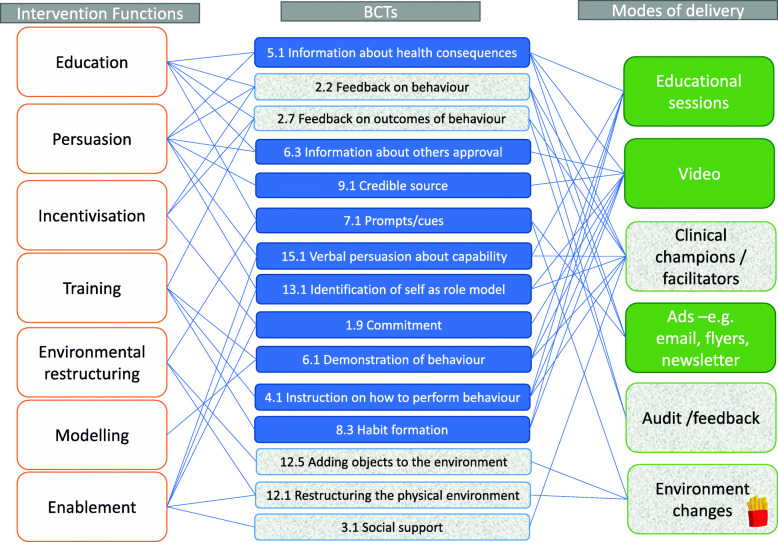
Logic map of intervention functions, BCTs, and modes of delivery for the ChIP implementation. Block colour indicates fully implemented BCTs and modes whereas shaded colour indicated partially implemented

The majority of action items were scored as partially or fully implemented (97.6%), considered high fidelity [24]. A summary of the complex relationship between intervention functions, BCTs, and modes of delivery has been collated in a logic map (Fig. 3). Filled boxes represent BCTs or modes that were fully implemented, and partially filled boxes represent BCTs or modes of delivery partially completed in the implementation. The main modes of delivery used were educational sessions, video, clinical champions, advertisements, audit and feedback, and environmental changes. As there is overlap with some of the BCTs addressing the same intervention functions, all intervention functions were still addressed even if not all modes were fully implemented.

Adaptations are integral to the implementation process and should be recorded, and there were no pre-determined restrictions to adaptations in this study [34]. Overall, the sites were very similar in the results. The smaller, rural site required more adaptations, needing a complete overhaul of the paging system to change to phone calls. Adaptations are a natural part of the implementation process; however, they need to be documented [35]. For example, the audits were discontinued; however, the audits were adapted to be part of the role of the existing CNCs. This adaptation is an example of an intervention becoming embedded in the system and leading to sustainability [36].

This study had limitations. It was difficult to find a framework to fit the acute care context impacting the rigour of analysis and conclusions. Frameworks exist for the evaluation of implementation fidelity [14]; however, the focus of these frameworks is often patient or client behaviours or the fidelity of delivery rather than the plan [9, 16, 37]. Evaluators need to be wary of using frameworks with an inappropriate focus, such as community compared to acute care [13]. The implementation of ChIP was within the hospital organisational system; to our knowledge, there are limited evaluations in this setting. The implementation strategies targeted the behaviour of staff involved in the activation or response to ChIP. There is a need for conceptual frameworks that evaluate implementation at the organisational level of healthcare to support implementation targeting staff change [7, 13]. Though a framework was used, the authors found the implementation outcomes were still not as specific as needed for the evaluation; future implementation plans should include a plan for how implementation strategy fidelity will be evaluated [25].

Retrospective completion of the scoring by the implementers relied somewhat on the memory of the implementers, which may be lacking. To counterbalance this, implementers were asked to supplement with documentation where possible (their log/audit records). Furthermore, the implementers completed the scoring separately so there would not be compromised results by relying on other opinions. Unfortunately, due to the small sample size, it was difficult to report on inter-rater agreement. The development of fidelity assessment criteria is another way to evaluate implementation strategy fidelity, which should be considered in implementation development rather than retrospective review [38].

It was a limitation that we could not fully report on the dose of the implementation strategy fidelity, which is the ‘proportion of intervention providers who received the implementation strategies’ [7], as it was difficult to evaluate the dose with confidence retrospectively. The dose would have included reporting how many staff members were reached with the various modes of delivery. However, with an implementation plan of this magnitude involving many layers of strategy, even prospectively, it would have been difficult to assess for dose. For example, the video was viewed by staff accessing the link themselves, but the video was also shown to large groups in education sessions, making it difficult to assess the true reach. Likewise, the flyers were posted in common areas, and other advertising materials were posted in the newsletter or emails. Even if we could identify staff who received the emails, it would be almost impossible to know the proportion of staff who engaged with these materials.

This study evaluates the implementation strategy fidelity for ChIP. This will help inform implementation in the emergency or multidisciplinary context. Planning for implementation includes consideration of the context of implementation and adaptations required at each site.

## Conclusion

This study evaluated two aspects of the fidelity of an implementation plan—the degree of implementation and the adaptations made. The findings indicated that implementation strategy fidelity might be assessed retrospectively from implementation documentation using a structured process evaluating behaviour change interventions. Adaptations to the implementation plan at one site may not be required at another site. A fidelity checklist developed during the implementation process may help implementers assess fidelity.

## Supplementary Information

**Additional file 1.** Implementation plan – redacted.

**Additional file 2.** StaRI checklist.

## Data Availability

The data supporting the conclusions of this article are included within the article. The individual data sets used in the evaluation are available from the corresponding author on reasonable request.

## Abbreviations

BCT: Behaviour change technique
BCW: Behaviour Change Wheel
ChIP: Chest Injury Protocol
CNC: Clinical nurse consultant
ED: Emergency department
ICU: Intensive care unit
