# Supplementary material for: Implementation strategy fidelity evaluation for a multidisciplinary Chest Injury Protocol (ChIP)
Source: Implement Sci Commun. 2021 Aug 10;2:86. doi: 10.1186/s43058-021-00189-8 (PMC8353870; doi:10.1186/s43058-021-00189-8)
Supplement: Supplementary file 1 — Additional file 1. Implementation plan – redacted. [file 43058_2021_189_MOESM1_ESM.docx]

***Chest injury protocol (ChIP)***

**IMPLEMENTATION STRATEGY**

**The Wollongong Hospital**

**Shoalhaven Memorial District Hospital**

Version *1.1*

*30/11/2017*

**VERSION HISTORY**

| **Version #** | **Implemented**  **By** | **Revision**  **Date** | **Approved**  **By** | **Approval**  **Date** | **Reason** |
| --- | --- | --- | --- | --- | --- |
| 1.0 | *Sarah Kourouche* | 6/11/17 | KC | 6/11/17 |  |
| 1.1 | Sarah Kourouche | 30/11/17 | KC | 30/11/17 | updated to do |
|  |  |  |  |  |  |
|  |  |  |  |  |  |

**Table of Contents**

[1 Introduction 4](#_Toc8811236)

[1.1 Purpose 4](#_Toc8811237)

[1.2 Intervention design 4](#_Toc8811238)

[1.3 The Intervention (Innovation): ChIP Overview 7](#_Toc8811239)

[1.4 Recipients 7](#_Toc8811240)

[1.4.1 Assumptions and Constraints 7](#_Toc8811241)

[1.5 Context 7](#_Toc8811242)

[2 Management Overview 7](#_Toc8811243)

[2.1 Description of Implementation 8](#_Toc8811244)

[2.2 Points-of-Contact 8](#_Toc8811245)

[2.3 Major Tasks 8](#_Toc8811246)

[2.4 Implementation Schedule 9](#_Toc8811247)

[2.5 Security and Privacy 10](#_Toc8811248)

[3 Implementation Support 10](#_Toc8811249)

[3.1 Resources 10](#_Toc8811250)

[3.1.1 Equipment 10](#_Toc8811251)

[3.1.2 Software 10](#_Toc8811252)

[3.2 Documentation 10](#_Toc8811253)

[3.3 Personnel 10](#_Toc8811254)

[3.4 Implementation Impact 11](#_Toc8811255)

[4.1 Evaluation 11](#_Toc8811256)

[4.2 Glossary 11](#_Toc8811257)

[APPENDIX A: ChIP POLICY 12](#_Toc8811258)

[15](#_Toc8811259)

[15](#_Toc8811260)

[APPENDIX B: Stakeholders* 16](#_Toc8811261)

[APPENDIX C: References 19](#_Toc8811262)

# Introduction

## 1.1 Purpose

The purpose of this document is to outline a plan for the implementation of a Chest Injury care bundle protocol (ChIP) for The Wollongong and Shoalhaven Memorial District Hospitals (TWH and SDMH respectively).

This document is part of a larger plan to more widely test and implement ChIP (Figure 1).

Figure 1: Overall process of ChIP testing and implementation

## ****1.2 Intervention design****

Table 1: Implementation Overview

| **Implementation Focus** | **Elements of the intervention** |
| --- | --- |
| **WHAT is being implemented**  **(Innovation)** | Evidence-based guidelines for blunt chest injury (ChIP)  Overarching aim: reduce pneumonia rates in blunt chest injury patients |
| **WHO is being targeted**  **(Recipients)** | Staff activating and responding to ChIP:  Surgical teams  Pain/Anaesthetic teams  Emergency department staff  Physiotherapists  Admissions staff  Ward staff |
| **WHERE**  **(Context)** | Acute health care setting: TWH and SDMH  Activated in emergency department  Local context assessment |
| **HOW**  **(Facilitation)** | Facilitation teams: external/internal expert and novice facilitators, clinical leaders, project manager and information specialist  Tailored facilitation support at the individual team level  Learning resources from Fisher and Paykel  Development of audit |

(Harvey & Kitson, 2016)

Behaviour change theories support this implementation. The Behaviour Change Wheel was used in conjunction with the Theoretical Domains Framework (TDF) (Figure 2) (Rycroft-Malone, 2004). This was to support the implementation further ensuring that intervention strategies would be appropriate for behaviour change to occur.

A survey of staff has been undertaken to inform the implementation process. The survey was based on the 14 domains of the TDF. Through mapping to the TDF, the survey has identified facilitators and barriers to implementation.

Intervention strategies were then identified in a step-by-step process to find intervention functions and policy changes that would need to occur for change to occur. The APEASE criteria (Affordability, Practicability, Effectiveness and cost-effectiveness, Acceptability, Side-effects and safety, and Equity) was used to identify if interventions were suitable for implementation. The resulting plan is in Table 2.


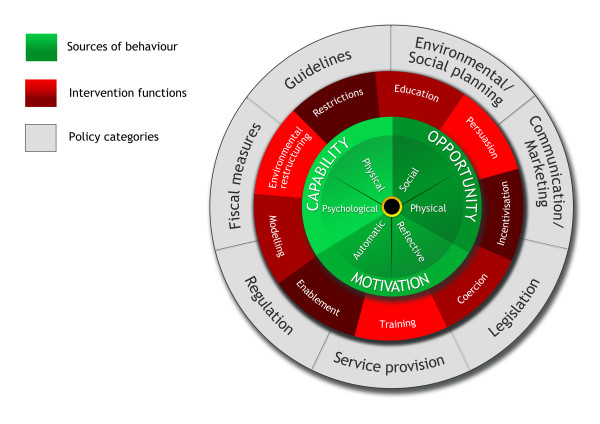


Figure 2. The behaviour change wheel with intervention functions and policy categories (Michie, van Stralen, & West, 2011)

Table 2.

| **Intervention functions** | **TDF components served by intervention functions** | **BCTs to deliver intervention functions** | **Policy categories through which BCTs can be delivered** | **Intervention strategy** |
| --- | --- | --- | --- | --- |
| **Education Persuasion, Incentivisation, Training, environmental restructuring, modelling, enablement,** | Physical skills  Knowledge  Memory, attention and decision processes  Professional/ social role and identity  Beliefs about consequences  Emotion  Environmental context and resources  Social influences | Information about health consequences  Feedback on behaviour  Prompts/cues  Feedback on outcome(s) of behaviour  Information about others’ approval  Credible source  Verbal persuasion about capability  Identification of self as role model  Incentive (outcome)  Commitment  Demonstration of behaviour  Instruction on how to perform behaviour  Habit formation  Adding objects to the environment  Restructuring the physical environment  Social Support | Communication/ marketing, guidelines, regulation, environmental/social planning | 1. Involve stakeholders in policy development 2. Open policy to comment 3. Set up of pager 4. In-services provided to ED and ward nurses and doctors, ICU regs, surgical registrars, physios, switch 5. Some of the in-services on HFNP by F&P 6. eMR icon added to FirstNet to identify CHIP 7. Guideline on intranet with easy search tool 8. Order Triflos 9. Video about CHIP 10. Newsletters – ED, ward, and surgical newsletters to inform of ChIP 11. Change champions self-elected from each area 12. Research nurses 13. Flyers with information with free chips to eat 14. Location of HFNP equipment, labelling of 15. Emails to all staff involved 16. Medical and nursing handover 17. Flyers in bathroom 18. Emails for managers 19. Clinical managers notice book |

## ****1.3 The Intervention: ChIP Overview****

ChIP is a care bundle of evidenced-based interventions for patients with a mild-moderate blunt chest injury (BCI). It aims to improve the outcomes of patients with blunt chest injury. ChIP has been used at another hospital resulting in a reduction in pneumonia by 56% (Curtis et al., 2016). The protocol has been refined with a literature review of interventions for blunt chest injury. The protocol can be found in Appendix A.

## ****1.4 Recipients****

The people involved in the implementation of this intervention are staff members who are involved in the activation and response to ChIP. This includes:

- Emergency nurses, doctors and clerical staff
- Surgical doctors
- Anaesthetics doctors
- Ward nurses caring for patients with BCI
- Physiotherapists
- Switchboard staff
- ICU liaison nurse
- ICU doctors
- Pain team

To ensure adequate involvement of recipients a survey of these staff was conducted.

### 1.4.1 Assumptions and Constraints

Stakeholders have been consulted before development of this plan, and it is assumed that their support will continue for the implementation process. All stakeholders will be consulted in the development process to increase the sense of ownership (Harvey & Kitson, 2016).

## ****1.5 Context****

The Wollongong and Shoalhaven Memorial District Hospitals (TWH and SDMH respectively) are the two sites where ChIP will be implemented. They are both within the same local health district (Illawarra-Shoalhaven).

# ****2 Management Overview****

The responsibility of management will lie with the ISLHD emergency clinical nurse consultants Professor Kate Curtis and Dr Belinda Munroe. Executive sponsors are Critical Care Co-Directors Michael Davis (medical) and Shanyn King (nursing), ISLHD Emergency services - medical director Thomas Carrigan and ISLHD Emergency services - service lead Orinda Jones.

Major tasks for management include:

- Communication with key stakeholders
- Review and approval of policy

## ****2.1 Description of Implementation****

ChIP will go live in both sites on the same day on 22^nd^ November 2017. The activation system through the communications team will be active from one week before (15/11/17).

Intervention Strategies are:

1. Involve stakeholders in policy development
2. Open policy to comment
3. Set up of pager
4. In-services provided to ED and ward nurses and doctors, ICU regs, surgical registrars, physios, switch
5. Some of the in-services on HFNP by F&P
6. eMR icon added to FirstNet to identify CHIP
7. Guideline on intranet with easy search tool
8. Order Triflos
9. Video about CHIP
10. Newsletters – ED, ward, and surgical newsletters to inform of ChIP
11. Change champions self-elected from each area
12. Research nurses as change champions
13. Flyers with information with free chips to eat
14. Location of HFNP equipment, labelling of
15. Emails to all staff involved
16. Flyers in bathroom
17. Emails for managers

## ****2.2 Points-of-Contact****

Stakeholders have been included from all areas involved, and a full list can be found in Appendix B.

## ****2.3 Major Tasks****

- Provide overall planning and coordination for the implementation
- Provide appropriate training for personnel
- Ensure that all manuals applicable to the implementation effort are available when needed
- Provide all needed technical assistance
- Schedule any special computer processing required for the implementation
- Perform site surveys before implementation
- Ensure that all prerequisites have been fulfilled before the implementation date
- Prepare site facilities for implementation
- Identifying and engaging with key stakeholders

## ****2.4 Implementation Schedule****

| **Task** | **Plan date by** | **Team member** | **Date completed** |
| --- | --- | --- | --- |
| Stakeholders approve policy | 14/8/17 | KC/BM | 20/11/17 |
| Policy submitted for general online comment | 15/10/17 | KC | 18/10/17 |
| Organise set up the ChIP pager | 1/11/17 | KC/BM | 21/11/17 |
| Organise dates and instructor (e.g. ICU liaison, research nurses) for In-services for CHIP:  Wards  ICU  ED  Surgical registrars  Anaesthetics  Physiotherapists | 20/11/17 | KC/BM | 31/10/17 |
| Development of training materials (PPT) | 15/11/17 | KC | 30/10/17 |
| Guideline on the intranet with easy search option | 1/11/17 | KC | In progress |
| Order Incentive spirometers | 15/11/17 | KC | Done |
| Education for HFNP from F&P   - Discuss with F&P - Organise times | 15/11/17 | KC | 20/11/17 |
| Flyers for ED | 1/11/17 | SK | 30/10/17 |
| Get chips packets to go with flyers in tea room | 15/11/17 | KC | 30/10/17 |
| Ensure HFNP marked with instructions | 1/11/17 | KC/BM | 23/10/17 |
| EMR icon | 1/11/17 | KC | 16/10/17 |
| Hire Casual research nurses | 1/11/17 | KC/BM | Contracts issued 31/10/17 |
| Research nurses informed of roles and can start championing process | 15/11/17 | KC | 10/11/17 |
| Video   - Get video footage - Put it together | 1/11 | KC/SK | Complete |
| Email to managers   - Checking if being implemented - Audit | 20/11/17 | SK/KC | progress |
| Email to staff   - Draft email for each area list them here | 20/11/17 | SK | 20/11/17 |
| Notice for Newsletters   - Surgical newsletter - EDs | 15/11/17 | SK | Surg NL – 30/10 |
| TWH clinical managers handover book notice | 20/11/17 | SK makes it  KC/BK put it in | 20/11/17 |

## ****2.5 Security and Privacy****

There are no breaches in security or privacy of patients or staff forseen by the implementation of ChIP.

# ****3 Implementation Support****

## ****3.1 Resources****

### **3.1.1 Equipment**

Limited high flow nasal prong (HFNP) sets are available. However, eight sets are being provided by Fisher and Paykel for use in the implementation which should suffice. However, not all staff are familiar with HFNP use and may need training in this area. Fisher and Paykel have kindly agreed to provide training support in this area.

### **3.1.2 Software**

The usual software will be used, i.e. FirstNet; however, as special Icon has been added for ChIP

## **3.2 Documentation**

Two protocols have been published relating to the implementation of ChIP.

1. ISLHD ChIP protocol (Appendix A) Link XXX
2. ISLHD HFNP protocol – Link XXX

## ****3.3 Personnel****

Facilitators

Facilitators will be from existing staff.

Research Nurses

Funding has been received as part of a larger project to support the implementation of ChIP from the HCF Research Foundation; some funds will be dedicated to the hiring of research nurses to support implementation.

Super users (Clinical champions)

Two super users in each area, need to have three years’ experience in the area and will receive training and first preference to attend sessions. They will assist on the floor, provide guidance, help with questions.

Instructors for in-services:

- Personnel from F&P
- ICU liaison
- Nurse educators
- Senior doctors
- Facilitators if needed
- Research nurses

## ****3.4****Implementation Impact

May have some initial increased requirements on staff. This will be reduced by an allocation of research nurses and clinical champions to provide extra facilitation.

## 4.1 Evaluation

The uptake of ChIP will be measured for six - twelve months post-implementation with a four-week gap as a buffer. This is part of a larger study and ethics will be sought. Furthermore, a second survey of staff may be done post-implementation for their evaluation if uptake levels are low.

## 4.2 Glossary

BCI – Blunt chest injury

ChIP – Chest Injury Protocol

ED – emergency department

HFNP – High flow Nasal Prongs

ISLHD – Illawarra Shoalhaven Local Health District

MRN – Medical record number

PT – Physiotherapist

# APPENDIX A: ChIP POLICY

1. POLICY STATEMENT

This policy describes the activation and response of a blunt chest injury pathway (ChIP), which promotes early intervention for patients who present to the Emergency Department (ED) with a blunt chest injury (proven or suspected rib fractures, or a painful chest injury)

2. AIMS

*Briefly state the aim of the policy that will help people understand this procedure.*

To describe the activation and response process for patients who present to the Emergency Department (ED) with a blunt chest injury.

3. TARGET AUDIENCE

*Include an outline of who the policy applies to.*

All clinicians who care for patients with blunt chest wall injury are responsible. In particular:

- Emergency Department (ED) medical and nursing staff
- Surgical registrars (covering ED)
- Physiotherapists
- Switch board staff
- Pain service / anaesthetics
- ICU liaison / resource nurses

4. RESPONSIBILITIES

*Outline clearly and succinctly the precise actions that are required of those responsible for enacting this policy.*

Members of this team will receive a message via their page to review patients with blunt chest wall injury who meet criteria. This policy also describes the recommended treatments to tailor for each patient dependent on their needs.

1. All patients with isolated blunt chest injury (radiological or clinical diagnosis) are to be considered for this pathway
2. The role of the ED Doctor and/or nurse is to assess the patient for likelihood of injury, attend to their immediate analgesia (+/- opiates), respiratory support needs and likelihood of admission before the ChIP page is activated (see Appendix 1)
3. To activate the ChIP page call 991 (TWH) or 9222 (SDMH), ChIP and patient MRN

1. The ChIP page will alert the on call Surgical registrar, Pain service (Anaesthetic Registrar TWH out of hours), Trauma CNC, ICU registrar, ICU Liaison Nurse, ASET (Aged Services Emergency Team) and Physiotherapist to enable early contact and optimal management. Where possible, the patient should be assessed by the responding clinicians within 60 minutes of the page activation.
2. When the required services are not available (eg after hours), the person carrying the pager should check for ChIP calls and review the patient as a priority when next on site.
3. The emergency or surgical registrar should arrange consultations with specialist teams such as Aged Care as needed.
4. All patients with proven or suspected rib fractures, or a painful chest injury requiring admission should be admitted under the surgical team on call.
5. A bundle of care including the following interventions should be charted, initiated and adapted for each patient according to their analgesic and respiratory support needs.

- Patient education on deep breathing and coughing and the following treatments
- Chest support pillow / splint (eg folded rolled towel)
- Incentive spirometry (triflow)
- Supplemental Oxygen, humidified via highflow nasal prongs – should commence at 50lt/min and titrated to an SpO2 goal in patients with pain not well controlled by oral analgesia and/or lung disease. A haemo/pneumothorax does not exclude the use of HFNP. Management of the haemo/pneumothorax should be discussed with the admitting consultant / fellow
- Analgesic regimen: PO Paracetamol and PO Oxycodone hydrochloride with Naloxone hydrochloride (Targin) or Oxycontin regularly if no contraindications/allergies & appropriate dose. PRN Oxycodone hydrochloride (Endone). NSAIDs should be considered in patients without contraindications
- If inadequate analgesia despite above, escalate early to pain service for PCA
- Regional anaesthesia techniques, such as an Epidural, paravertebral or intercostal block, should be considered on a case by case basis. In particular those patients with: 3 or more rib fractures and/or age greater than 55 years. Additional consideration and factors that may infer patient benefit to those with (or without) the above factors include flail segment/s, underlying lung disease, history of smoking
- Aperients and anti-emetics
- Early mobilisation as clinically appropriate

1. Patient should be admitted to a ward with HFNP capability. If the patient has increasing FiO2 requirements to maintain respiratory function and observations between the flags, they should have an ICU review and admission.
2. Weaning of analgesia and HFNP should be conducted on individual patient needs. The patient should be discharged when their pain is well controlled with oral analgesia, their respiratory function has been optimised and any other factors (i.e. mobility and any medical issues that may have precipitated a fall) are resolved
3. ChIP patients should be followed up by their GP within 3 days and their analgesia. Discharge letter should include instruction on this for the GP.
4. Patients and their family should be educated throughout their admission and prior to discharge on the importance of continuing with regular analgesia as prescribed, signs of deterioration and advice to represent if necessary. The following fact sheet should be provided and explained to the patient <https://www.aci.health.nsw.gov.au/__data/assets/pdf_file/0010/294337/Fractured_Ribs_Patient_Factsheet_2015.pdf>

5. DEFINITIONS

HFNP: High Flow nasal prong

Blunt chest injury: Proven or suspected rib fractures, or a painful chest injury

Bundle of care: A group of evidence based interventions administered for specific conditions

6. DOCUMENTATION

*None required.*

7. AUDIT

The activation and appropriate use of the protocol will be evaluated by a formal research project, then ongoing monitoring will be monitored by the existing trauma service quality improvement program. Regular reports are conducted, feedback is provided where appropriate

REFERENCES

1. Testerman GM. Adverse outcomes in younger rib fracture patients. South Med J. 2006;99(4):335-339.

2. Holcomb JB, McMullin NR, Kozar RA, Lygas MH, Moore FA. Morbidity from rib fractures increases after age 45. Journal of the American College of Surgeons. 2003;196(4):549-555.

3. Kent R, Woods W, Bostrom O. Fatality risk and the presence of rib fractures. Paper presented at: Annals of Advances in Automotive Medicine - 52nd Annual Scientific Conference2008.

4. Bulger EM, Arneson MA, Mock CN, Jurkovich GJ. Rib fractures in the elderly. Journal of Trauma - Injury, Infection and Critical Care. 2000;48(6):1040-1046.

5. Barnea Y, Kashtan H, Skornick Y, Werbin N. Isolated rib fractures in elderly patients: Mortality and morbidity. Canadian Journal of Surgery. 2002;45(1):43-46.

6. Elmistekawy E, Hammad AA. Isolated rib fractures in geriatric patients. Annals of Thoracic Medicine. 2007;2(4):166-168.

7. Bansidhar BJ, Lagares-Garcia JA, Miller SL. Clinical rib fractures: are follow-up chest X-rays a waste of resources? The American surgeon. May 2002;68(5):449-453.

8. Lee RB, Bass SM, Morris JA, Jr., MacKenzie EJ. Three or more rib fractures as an indicator for transfer to a Level I trauma center: a population-based study. Journal of Trauma-Injury Infection & Critical Care. 1990;30(6):689-694.

9. Battle CE, Hutchings H, Evans PA. Risk factors that predict mortality in patients with blunt chest wall trauma: a systematic review and metaanalysis. Injury. 2012;43(1):8-17.

10. Curtis K, Asha S, et al. ChIP: An early activation protocol for isolated blunt chest injury improves outcomes. Australasian Journal of Emergency Nursing. 2016

APPENDIX 1

Initial Assessment
Assess particularly for pain and ability to deep breath/cough/SpO2 + CXR and Analgesia

The CHIP page will alert:
- Surgical team
- Pain team (within hrs)/ Anaesthetics (after hrs)
- Physiotherapy (PT)
- ICU Registrar

- ICU Liaison Nurse

- Trauma CNC
- ASET

Red Flags
Age > 55yrs
Respiratory history
Respiratory compromise
 (eg: ↑WOB; ↑RR; ↓SpO2
≥3 rib #s)

Analgesic regime:
- Oral: Targin, Paracetamol, NSAIDs
PRN Endone
- IV: Consider PCA
- Consider IC Block/ Paravertebral Block/ Epidural

Clearly document HFNP settings as well as SpO2 goal in patient’s notes.

Referrals to consider prior to transfer:
Low threshold for ICU admission. GenMed if needed

Don’t forget aperients

Complication Prevention:
Early Clearance to Mobilise
Patient education

DB&C

Regular and PRN analgesia
Incentive spirometry
Support/splint pillows

If patient for admission….
AMO1= Surgery. AMO2 as appropriate (eg GenMed)

Appropriate Bed Allocation (HFNP capability +/- ICU)

CALL 991 or 9222 (site specific)

ACTIVATE CHIP AND Provide MRN
Patient to be reviewed by on call Surgical Registrar to determine if admission is required.

Discharge Planning from ward:
Wean HFNP/Analgesia (per patient condition)
Discharge home when pain well controlled & respiratory function optimised
Patient / family education to include return to ED if breathing or pain worsens
GP f/u within 3 days

Respiratory Adjuncts:
Consider HFNP:
Start at 50L Flow/ Fio2 30-40%
(Does not exclude HTx/PTx – but discuss with surg fellow)

Re-assessment
Not able to deep breathe/cough; Ongoing pain (at least 30mins post initial analgesia)

# APPENDIX B: Stakeholders*

| **Key stakeholders** | **Rationale** |
| --- | --- |
| **District level** |  |
| **Facilitators** | Facilitate the implementation process.  Clinical champion, protocol development, local |
| **Site Implementation Representative SDMH** | Facilitate the implementation process.  Clinical champion, protocol development, local |
| **Business sponsors** | Representative from HCF – funding body of overarching project |
|  | Representative from Fisher and Paykel- funding of HFNC machines and will provide support |
| **Executive sponsors** | Critical Care Co-Directors.  Executive support |
|  |  |
|  | ISLHD Emergency services - medical director  Executive support |
|  | Executive support  Determine appropriate ward allocation and engage with NUMs |
| **DDON / DONs at each site** | Exec endorsement |
|  |  |
| **Surgical** | Support required as patients will be admitted under them |
|  |  |
| **ED District Nurse educator** | Clinical champion, enforce with staff, Protocol development, local knowledge |
| **Physiotherapy District manager** | Representative of physiotherapists |
| **Trauma committee** |  |
|  | Exec endorsement |
|  |  |
|  |  |
| **Research Office** | Research support, smoothness of ethics process |
| **Switchboard** | Support implementation |
| **TWH** |  |
| **Activators** |  |
| **ED Director** | Clinical champion, enforce with staff |
| **ED medical staff** |  |
| **ED NUM** | Clinical champion, enforce with staff |
| **ED Clinical NUM / patient flow** | Clinical champion, enforce with staff, Protocol development, local knowledge |
|  |  |
| **ED CNC** |  |
| **ED CNE** | Clinical champion, enforce with staff |
| **ED nursing staff** |  |
| **Responders** |  |
| **Surgeons** |  |
|  |  |
|  |  |
| **Anaesthetics / pain team** | Patients will be reviewed by them (in and out of hours). Need agreement |
| **Physiotherapy manager** | Support required as patients will be reviewed by them |
|  |  |
| **ICU Director** | Support required as patients will potentially be reviewed by them |
|  |  |
| **ICU liaison nurse / Clinical Resource nurse / ICU NUM** | Patient should be reviewed by them |
| **Support** |  |
| **DON** |  |
| **ED equipment officer** |  |
| **Switch board** |  |
| **Trauma service** |  |
| **Bed managers / AH** | Appropriate bed allocation |
| **CNC for surgery** | Patient should be reviewed by them |
| **Ward NUMs** |  |
| **SDMH** |  |
| **Activators** |  |
| **ED Director** | Clinical champion, enforce with staff |
| **ED NUM** | Clinical champion, enforce with staff |
| **ED medical staff** |  |
| **ED CNC** |  |
| **ED CNE** |  |
| **ED nursing staff** |  |
|  |  |
| **ED Clinical NUM / patient flow** | Clinical champion, enforce with staff, Protocol development, local knowledge |
|  |  |
| **Responders** |  |
| **Surgery** | Support required as patients will be admitted under them |
|  |  |
| **Anaesthetics / Pain team** |  |
|  |  |
| **Physiotherapy** | Support required as patients will be reviewed by them |
| **ICU** | Support required as patients will potentially be reviewed by them |
|  |  |
| **Support** |  |
| **DON** |  |
| **ED equipment officer** |  |
| **Switch board** | Support Implementation |
| **Trauma service** | N/A |
| **Bed managers / AH** | Appropriate bed allocation |
| **Ward NUMs** |  |

*Columns with names, contact details and status have been removed to protect confidentiality.

# APPENDIX C: References

Curtis, K., Asha, S. E., Unsworth, A., Lam, M., Goldsmith, H., Langcake, M., & Dwyer, D. (2016). ChIP: An early activation protocol for isolated blunt chest injury improves outcomes, a retrospective cohort study. *Australasian Emergency Nursing Journal, 19*(3), 127-132. doi:10.1016/j.aenj.2016.06.002

Harvey, G., & Kitson, A. (2016). PARIHS revisited: from heuristic to integrated framework for the successful implementation of knowledge into practice. *IMPLEMENTATION SCIENCE, 11*(1), 33. doi:10.1186/s13012-016-0398-2

Michie, S., van Stralen, M. M., & West, R. (2011). The behaviour change wheel: a new method for characterising and designing behaviour change interventions. *Implementation science : IS, 6*(1), 42-42. doi:10.1186/1748-5908-6-42

Rycroft-Malone, J. (2004). The PARIHS framework - A framework for guiding the implementation of evidence-based practice. *Journal of Nursing Care Quality, 19*(4), 297-304. doi:10.1097/00001786-200410000-00002

Stetler, C. B., Damschroder, L. J., Helfrich, C. D., & Hagedorn, H. J. (2011). A Guide for applying a revised version of the PARIHS framework for implementation. *Implementation science : IS, 6*(1), 99-99. doi:10.1186/1748-5908-6-99
